# 5-[(4-Fluoro­anilino)meth­yl]-6-methyl-*N*-(4-methyl­phen­yl)-2-phenyl­pyrimidin-4-amine

**DOI:** 10.1107/S160053681203783X

**Published:** 2012-09-12

**Authors:** Jerzy Cieplik, Marcin Stolarczyk, Iwona Bryndal, Tadeusz Lis

**Affiliations:** a Department of Organic Chemistry, Medical Academy, 9 Grodzka St, 50-137 Wrocław, Poland; b Faculty of Chemistry, University of Wrocław, 14 Joliot-Curie St, 50-383 Wrocław, Poland; c Department of Bioorganic Chemistry, Faculty of Engineering and Economics, Wrocław University of Economics, 118/120 Komandorska St, 53-345 Wrocław, Poland

## Abstract

In the title compound, C_25_H_23_FN_4_, the pyrimidine ring makes dihedral angles of 11.3 (2), 24.5 (2) and 70.1 (2)° with the phenyl and two benzene rings, and the mol­ecular conformation is stabilized by an intra­molecular N—H⋯N hydrogen bond with an *S*(6) ring motif. In the crystal, a pair of weak C—H⋯F hydrogen bonds link two mol­ecules into an inversion dimer with an *R*_2_^2^(16) motif. In the dimer, there is also an inter­molecular π–π stacking inter­action [centroid–centroid distance = 3.708 (4) Å] between the fluorinated benzene rings. The dimers are further linked by a C—H⋯π inter­action, so forming a column along the *c* axis.

## Related literature

For the anti­bacterial activity of 6-methyl-2-phenyl-5-substituted pyrimidine derivatives, see: Cieplik *et al.* (2003 ▶, 2008 ▶); Cieplik, Stolarczyk *et al.* (2011 ▶). For related structures, see: Cieplik *et al.* (2006 ▶, 2012 ▶); Cieplik, Pluta *et al.* (2011 ▶).
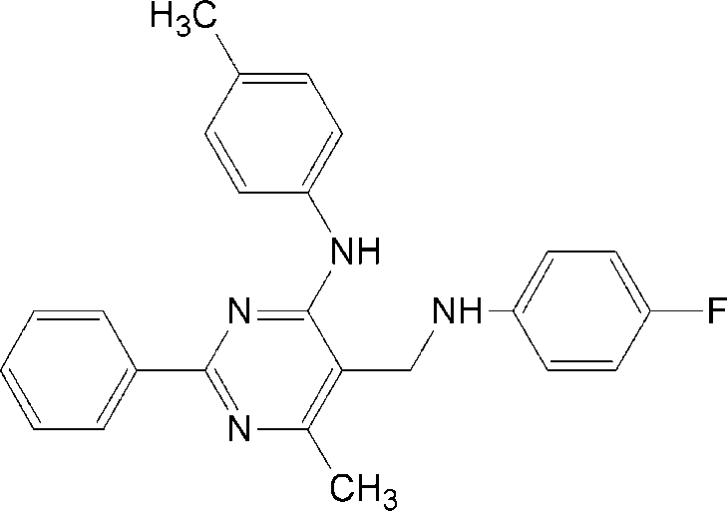


## Experimental

### 

#### Crystal data

C_25_H_23_FN_4_
*M* *_r_* = 398.47
Triclinic, 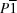

*a* = 8.306 (4) Å
*b* = 10.070 (4) Å
*c* = 12.234 (5) Å
α = 88.78 (4)°
β = 89.12 (4)°
γ = 82.75 (5)°
*V* = 1014.8 (8) Å^3^
*Z* = 2
Mo *K*α radiation
μ = 0.09 mm^−1^
*T* = 85 K
0.36 × 0.18 × 0.14 mm

#### Data collection

Oxford Xcalibur PX diffractometer with Onyx CCD
17245 measured reflections
8461 independent reflections
4334 reflections with *I* > 2σ(*I*)
*R*_int_ = 0.033

#### Refinement

*R*[*F* ^2^ > 2σ(*F* ^2^)] = 0.049
*wR*(*F* ^2^) = 0.093
*S* = 1.00
8461 reflections
279 parameters
H atoms treated by a mixture of independent and constrained refinement
Δρ_max_ = 0.38 e Å^−3^
Δρ_min_ = −0.31 e Å^−3^

### 

Data collection: *CrysAlis CCD* (Oxford Diffraction, 2007 ▶); cell refinement: *CrysAlis CCD*; data reduction: *CrysAlis RED* (Oxford Diffraction, 2007 ▶); program(s) used to solve structure: *SHELXS97* (Sheldrick, 2008 ▶); program(s) used to refine structure: *SHELXL97* (Sheldrick, 2008 ▶); molecular graphics: *XP* in *SHELXTL* (Sheldrick, 2008 ▶); software used to prepare material for publication: *SHELXL97*.

## Supplementary Material

Crystal structure: contains datablock(s) I, global. DOI: 10.1107/S160053681203783X/is5187sup1.cif

Structure factors: contains datablock(s) I. DOI: 10.1107/S160053681203783X/is5187Isup2.hkl

Supplementary material file. DOI: 10.1107/S160053681203783X/is5187Isup3.cml

Additional supplementary materials:  crystallographic information; 3D view; checkCIF report

## Figures and Tables

**Table 1:** Hydrogen-bond geometry (Å, °) *Cg*1 is the centroid of the N1/C2/N3/C4–C6 ring.

*D*—H⋯*A*	*D*—H	H⋯*A*	*D*⋯*A*	*D*—H⋯*A*
N4—H4⋯N5	0.869 (11)	2.294 (11)	2.9820 (18)	136.2 (9)
C57—H571⋯F5^i^	0.99	2.54	3.440 (2)	151
C57—H572⋯*Cg*1^ii^	0.99	2.60	3.467 (7)	147

Symmetry codes: (i) 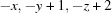; (ii) 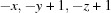.

**Fig. 1.**
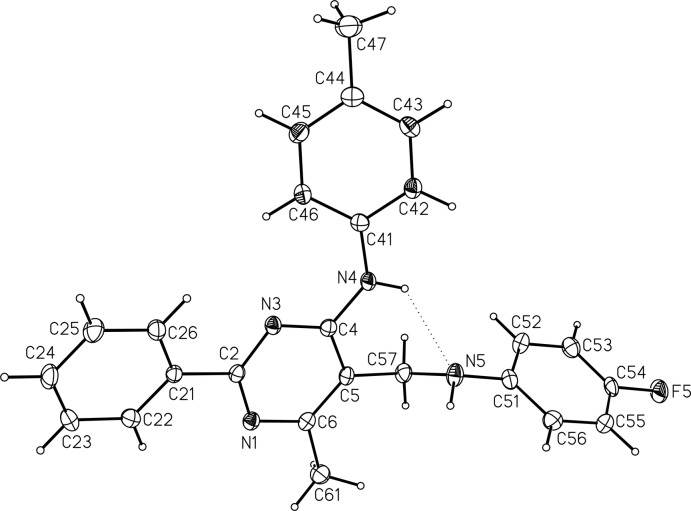
The molecule of title compound, showing the atom-numbering scheme. Displacements ellipsoids are drawn at the 50% probability level and H atoms are shown as small spheres of arbitrary radii. The dotted line indicates the intramolecular N—H···N hydrogen bond.

**Fig. 2.**
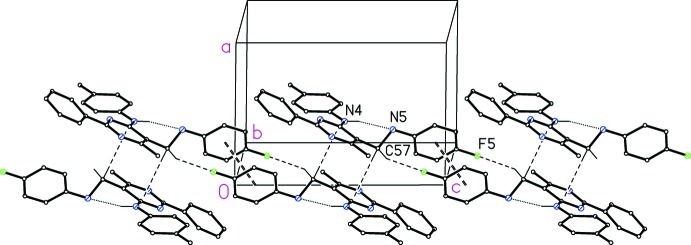
Part of the crystal structure of title compound, showing the intermolecular C—H···F, C—H···π (dashed lines) and π–π stacking interactions (double dashed lines). Dotted lines indicate intramolecular N—H···N interactions. H atoms not involved in hydrogen bonding have been omitted for clarity.

## supplementary crystallographic information

### Comment

The pyrimidine core attracts attention as natural products and pharmacologically active compounds. In our ongoing research on an immunomodulating agent, we have synthesized some of 6-methyl-2-phenyl-5-substituted pyrimidine derivatives and their antibacterial and antifungal activities have been reported (Cieplik *et al.*, 2003, 2008; Cieplik, Stolarczyk *et al.*, 2011). As part of our study, we previously characterized structures of polymorphic forms of *N*-(4-chlorophenyl)-5-[(4-chlorophenyl)aminomethyl]-6-methyl-2-phenylpyrimidin-4-amine (Cieplik *et al.*, 2006) and *N*-(2-fluorophenyl)-5-[(4-alloxyphenyl)aminomethyl]-6-methyl-2-phenylpyrimidin-4-amines (Cieplik, Pluta *et al.*, 2011; Cieplik *et al.*, 2012). In the continuation of our search for more potent antibacterial agent, the title compound, 5-[(4-fluoroanilino)methyl]-6-methyl-2-*N*-(4-methylphenyl)-phenylpyrimidin-4-amine, was also prepared.

The title compound crystallizes in *P*1 space group, with one molecule in the asymmetric unit (Fig. 1). There is an intramolecular N—H···N hydrogen bond between N4—H4 and N5, which closes a six-membered ring (Table 1). The N4···N5 distance [2.982 (2) Å] is longer than its conterparts in other similar compound (Cieplik, Pluta *et al.*, 2011; Cieplik, Stolarczyk *et al.*, 2011), compared with 2.940 (3) Å for the polymorphic form of *N*-(4-chlorophenyl)-5-[(4-chlorophenyl)aminomethyl]-6-methyl-2-phenylpyrimidin-4-amine (denoted as I*a*, Cieplik *et al.*, 2006). The conformation of the title molecule is best defined by dihedral angles formed between the pyrimidine ring and the planes of the phenyl ring attached to atom C2 and two other aryl rings of the (4-methylphenyl)amino and the (4-fluorophenyl)aminomethyl groups attached, respectively, to atoms C4 and C5 of the pyrimidine ring. These dihedral angles are 11.3 (2), 24.5 (2) and 70.1 (2)°, respectively.

The N—H···N hydrogen bonds involving the amine atom N5 as a donor are commonly observed in previously described structures (Cieplik *et al.*, 2006, 2012; Cieplik, Pluta *et al.*, 2011). However, in the structure of the title compound, atom H5 is not involved in any interactions. The crystal structure of the title compound is stabilized by weak C—H···F, C—H···π and π–π stacking interactions. The molecules are linked by a C—H···F interaction involving atom C57 as a donor and atom F5 (-*x*, -*y* + 1, -*z* + 2) as an acceptor. This results in the formation of an inversion dimer with an *R*^2^_2_(16) ring motif. Between aryl rings of (4-fluorophenyl)aminomethyl groups of molecules forming the dimer there is also an aromatic π–π stacking interaction. The distance between the centroids of C51–C56 ring at (*x*, *y*, *z*) and (-*x*, -*y* + 1, -*z* + 2) is 3.708 (4) Å, and the interplanar spacing and the centroid offset are 3.429 (4) Å and 1.41 Å, respectively. Additionally, the C57—H572 group acts as a donor of C—H···π(arene) interaction to the pyrimidine N1/C2/N3/C4–C6 ring (-*x*, -*y* + 1, -*z* + 1). The combination of C—H···F and C—H···π interactions generates a column running along the [001] direction (Fig. 2).

### Experimental

The title compound was obtained by adopting the procedure described previously by Cieplik *et al.* (2003). 4 g (0.0125 mmol) of 5-(chloromethyl)-6-methyl-*N*-(4-methylphenyl)-2-phenylpyrimidin-4-amine was dissolved in 50 ml of chloroform, and 2 g of 4-fluoroaniline was added. The reaction mixture was refluxed for 4 h with vigorous stirring, then was cooled and poured into 100 ml of water. The aqueous solution was extracted three times with chloroform (50 ml). The combined chloroform phases were dried over MgSO_4_, filtered and concentrated under vacuum. The oily residue was purified by column chromatography on silica gel (200–400 mesh) using CHCl_3_ as the eluent and by crystallization from methanol to give single crystals (yield: 3.74 g, 76.0%, m.p. 423–425 K).

### Refinement

The N-bonded H atoms were found in a difference Fourier map and their positions were refined with *U*_iso_(H) = 1.2*U*_eq_(N). The remaining H atoms were treated as riding on their carrier atoms, with C—H distances in the range 0.95–0.99 Å, and with *U*_iso_(H) = 1.2*U*_eq_(C), except methyl groups where *U*_iso_(H) = 1.5*U*_eq_(C).

### Crystal data

**Table:**

C_25_H_23_FN_4_	*Z* = 2
*M**_r_* = 398.47	*F*(000) = 420
Triclinic, *P*1	*D*_x_ = 1.304 Mg m^−^^3^
Hall symbol: -P 1	Melting point: 424 K
*a* = 8.306 (4) Å	Mo *K*α radiation, λ = 0.71073 Å
*b* = 10.070 (4) Å	Cell parameters from 9708 reflections
*c* = 12.234 (5) Å	θ = 4.8–35.0°
α = 88.78 (4)°	µ = 0.09 mm^−^^1^
β = 89.12 (4)°	*T* = 85 K
γ = 82.75 (5)°	Needle block, light yellow
*V* = 1014.8 (8) Å^3^	0.36 × 0.18 × 0.14 mm

### Data collection

**Table:**

Oxford Xcalibur PX diffractometer with Onyx CCD	4334 reflections with *I* > 2σ(*I*)
Radiation source: normal-focus sealed tube	*R*_int_ = 0.033
Graphite monochromator	θ_max_ = 35.0°, θ_min_ = 4.8°
φ and ω scans	*h* = −11→13
17245 measured reflections	*k* = −15→16
8461 independent reflections	*l* = −19→19

### Refinement

**Table:**

Refinement on *F*^2^	Primary atom site location: structure-invariant direct methods
Least-squares matrix: full	Secondary atom site location: difference Fourier map
*R*[*F*^2^ > 2σ(*F*^2^)] = 0.049	Hydrogen site location: inferred from neighbouring sites
*wR*(*F*^2^) = 0.093	H atoms treated by a mixture of independent and constrained refinement
*S* = 1.00	*w* = 1/[σ^2^(*F*_o_^2^) + (0.030*P*)^2^] where *P* = (*F*_o_^2^ + 2*F*_c_^2^)/3
8461 reflections	(Δ/σ)_max_ = 0.001
279 parameters	Δρ_max_ = 0.38 e Å^−^^3^
0 restraints	Δρ_min_ = −0.31 e Å^−^^3^

### Special details

**Table:**

Geometry. All e.s.d.'s (except the e.s.d. in the dihedral angle between two l.s. planes) are estimated using the full covariance matrix. The cell e.s.d.'s are taken into account individually in the estimation of e.s.d.'s in distances, angles and torsion angles; correlations between e.s.d.'s in cell parameters are only used when they are defined by crystal symmetry. An approximate (isotropic) treatment of cell e.s.d.'s is used for estimating e.s.d.'s involving l.s. planes.
Refinement. Refinement of *F*^2^ against ALL reflections. The weighted *R*-factor *wR* and goodness of fit *S* are based on *F*^2^, conventional *R*-factors *R* are based on *F*, with *F* set to zero for negative *F*^2^. The threshold expression of *F*^2^ > σ(*F*^2^) is used only for calculating *R*-factors(gt) *etc*. and is not relevant to the choice of reflections for refinement. *R*-factors based on *F*^2^ are statistically about twice as large as those based on *F*, and *R*- factors based on ALL data will be even larger.

### Fractional atomic coordinates and isotropic or equivalent isotropic displacement parameters (Å^2^)

**Table:**

	*x*	*y*	*z*	*U*_iso_*/*U*_eq_	
N1	0.10445 (10)	0.79035 (8)	0.41851 (7)	0.01576 (19)	
C2	0.20919 (12)	0.71729 (10)	0.35266 (8)	0.0140 (2)	
C21	0.25288 (12)	0.78216 (10)	0.24794 (8)	0.0146 (2)	
C22	0.16726 (13)	0.90318 (10)	0.21321 (9)	0.0202 (2)	
H22	0.0795	0.9441	0.2565	0.024*	
C23	0.20876 (14)	0.96450 (11)	0.11618 (9)	0.0249 (3)	
H23	0.1488	1.0466	0.0930	0.030*	
C24	0.33733 (13)	0.90659 (11)	0.05289 (9)	0.0232 (3)	
H24	0.3651	0.9485	−0.0139	0.028*	
C25	0.42520 (13)	0.78748 (11)	0.08714 (9)	0.0223 (2)	
H25	0.5144	0.7482	0.0443	0.027*	
C26	0.38325 (13)	0.72516 (10)	0.18414 (8)	0.0185 (2)	
H26	0.4438	0.6432	0.2071	0.022*	
N3	0.28386 (10)	0.59329 (8)	0.37320 (7)	0.01478 (18)	
C4	0.25062 (12)	0.53878 (10)	0.47043 (8)	0.0142 (2)	
N4	0.32494 (10)	0.41352 (8)	0.49809 (7)	0.01661 (19)	
H4	0.3281 (13)	0.3965 (11)	0.5680 (9)	0.020*	
C41	0.42036 (12)	0.31806 (10)	0.43411 (8)	0.0152 (2)	
C42	0.50900 (12)	0.21177 (10)	0.49004 (9)	0.0173 (2)	
H42	0.5058	0.2087	0.5677	0.021*	
C43	0.60151 (12)	0.11080 (10)	0.43404 (9)	0.0191 (2)	
H43	0.6628	0.0406	0.4741	0.023*	
C44	0.60703 (12)	0.10953 (10)	0.32041 (9)	0.0197 (2)	
C47	0.70423 (14)	−0.00354 (12)	0.26030 (10)	0.0290 (3)	
H473	0.7735	−0.0592	0.3120	0.043*	
H472	0.7721	0.0333	0.2040	0.043*	
H471	0.6303	−0.0582	0.2259	0.043*	
C45	0.51525 (13)	0.21488 (10)	0.26533 (9)	0.0207 (2)	
H45	0.5153	0.2158	0.1877	0.025*	
C46	0.42383 (12)	0.31843 (10)	0.31983 (8)	0.0183 (2)	
H46	0.3638	0.3893	0.2797	0.022*	
C5	0.14131 (12)	0.60620 (10)	0.54702 (8)	0.0146 (2)	
C57	0.09810 (12)	0.53600 (11)	0.65109 (8)	0.0181 (2)	
H571	0.0099	0.5926	0.6897	0.022*	
H572	0.0578	0.4507	0.6332	0.022*	
N5	0.23857 (11)	0.50820 (9)	0.72329 (7)	0.0198 (2)	
H5	0.2891 (13)	0.5843 (11)	0.7280 (9)	0.024*	
C51	0.20720 (12)	0.45585 (10)	0.82908 (8)	0.0171 (2)	
C52	0.11769 (13)	0.34826 (11)	0.84171 (9)	0.0210 (2)	
H52	0.0775	0.3099	0.7790	0.025*	
C53	0.08662 (13)	0.29654 (11)	0.94486 (9)	0.0215 (2)	
H53	0.0245	0.2239	0.9535	0.026*	
F5	0.11487 (8)	0.30347 (6)	1.13620 (5)	0.02904 (17)	
C54	0.14766 (13)	0.35259 (10)	1.03413 (8)	0.0197 (2)	
C55	0.23850 (13)	0.45671 (11)	1.02485 (9)	0.0228 (2)	
H55	0.2808	0.4927	1.0880	0.027*	
C56	0.26771 (13)	0.50879 (11)	0.92156 (9)	0.0215 (2)	
H56	0.3298	0.5815	0.9140	0.026*	
C6	0.07285 (12)	0.73425 (10)	0.51736 (8)	0.0154 (2)	
C61	−0.03893 (13)	0.82245 (11)	0.59100 (9)	0.0211 (2)	
H611	−0.1495	0.7992	0.5855	0.032*	
H612	−0.0375	0.9164	0.5687	0.032*	
H613	−0.0025	0.8093	0.6667	0.032*	

### Atomic displacement parameters (Å^2^)

**Table:**

	*U*^11^	*U*^22^	*U*^33^	*U*^12^	*U*^13^	*U*^23^
N1	0.0161 (4)	0.0172 (4)	0.0143 (4)	−0.0035 (3)	0.0008 (4)	0.0001 (4)
C2	0.0141 (5)	0.0158 (5)	0.0124 (5)	−0.0035 (4)	−0.0012 (4)	0.0000 (4)
C21	0.0174 (5)	0.0145 (5)	0.0125 (5)	−0.0040 (4)	−0.0001 (4)	−0.0001 (4)
C22	0.0250 (6)	0.0162 (5)	0.0187 (6)	0.0000 (4)	0.0028 (5)	0.0003 (4)
C23	0.0348 (7)	0.0175 (6)	0.0213 (6)	0.0001 (5)	0.0004 (5)	0.0055 (5)
C24	0.0314 (7)	0.0247 (6)	0.0150 (6)	−0.0107 (5)	0.0014 (5)	0.0041 (5)
C25	0.0221 (6)	0.0271 (6)	0.0177 (6)	−0.0042 (5)	0.0058 (5)	0.0010 (5)
C26	0.0199 (6)	0.0186 (5)	0.0162 (5)	−0.0006 (4)	0.0014 (4)	0.0017 (4)
N3	0.0156 (4)	0.0157 (4)	0.0135 (4)	−0.0038 (3)	0.0003 (3)	0.0012 (3)
C4	0.0147 (5)	0.0149 (5)	0.0138 (5)	−0.0042 (4)	−0.0007 (4)	−0.0001 (4)
N4	0.0215 (5)	0.0166 (5)	0.0109 (4)	0.0002 (4)	0.0017 (4)	0.0023 (4)
C41	0.0152 (5)	0.0138 (5)	0.0169 (5)	−0.0040 (4)	0.0016 (4)	0.0005 (4)
C42	0.0192 (5)	0.0178 (5)	0.0156 (5)	−0.0052 (4)	−0.0016 (4)	0.0012 (4)
C43	0.0176 (5)	0.0160 (5)	0.0236 (6)	−0.0022 (4)	−0.0036 (5)	0.0017 (4)
C44	0.0181 (5)	0.0182 (5)	0.0234 (6)	−0.0042 (4)	0.0015 (5)	−0.0026 (5)
C47	0.0305 (7)	0.0249 (6)	0.0308 (7)	0.0006 (5)	−0.0008 (5)	−0.0067 (5)
C45	0.0249 (6)	0.0218 (6)	0.0159 (6)	−0.0048 (5)	0.0018 (5)	−0.0018 (4)
C46	0.0210 (6)	0.0180 (5)	0.0156 (5)	−0.0016 (4)	−0.0014 (4)	0.0028 (4)
C5	0.0149 (5)	0.0180 (5)	0.0115 (5)	−0.0040 (4)	0.0002 (4)	0.0008 (4)
C57	0.0190 (6)	0.0212 (5)	0.0137 (5)	−0.0020 (4)	0.0031 (4)	0.0019 (4)
N5	0.0186 (5)	0.0262 (5)	0.0154 (5)	−0.0067 (4)	−0.0006 (4)	0.0043 (4)
C51	0.0148 (5)	0.0206 (5)	0.0148 (5)	0.0018 (4)	0.0020 (4)	0.0033 (4)
C52	0.0225 (6)	0.0257 (6)	0.0151 (6)	−0.0042 (5)	−0.0026 (5)	0.0000 (5)
C53	0.0222 (6)	0.0212 (6)	0.0210 (6)	−0.0032 (4)	0.0015 (5)	0.0044 (5)
F5	0.0398 (4)	0.0301 (4)	0.0151 (3)	0.0018 (3)	0.0043 (3)	0.0068 (3)
C54	0.0239 (6)	0.0205 (6)	0.0122 (5)	0.0055 (4)	0.0039 (4)	0.0053 (4)
C55	0.0299 (6)	0.0221 (6)	0.0155 (6)	0.0007 (5)	−0.0028 (5)	−0.0020 (5)
C56	0.0243 (6)	0.0195 (6)	0.0209 (6)	−0.0030 (4)	0.0002 (5)	0.0000 (5)
C6	0.0137 (5)	0.0188 (5)	0.0140 (5)	−0.0035 (4)	0.0001 (4)	−0.0011 (4)
C61	0.0224 (6)	0.0212 (6)	0.0190 (6)	−0.0009 (4)	0.0057 (5)	−0.0012 (4)

### Geometric parameters (Å, º)

**Table:**

N1—C2	1.3377 (14)	C47—H473	0.9800
N1—C6	1.3598 (14)	C47—H472	0.9800
C2—N3	1.3427 (14)	C47—H471	0.9800
C2—C21	1.4865 (15)	C45—C46	1.3867 (16)
C21—C22	1.3928 (16)	C45—H45	0.9500
C21—C26	1.3955 (16)	C46—H46	0.9500
C22—C23	1.3853 (16)	C5—C6	1.3853 (15)
C22—H22	0.9500	C5—C57	1.5037 (15)
C23—C24	1.3845 (17)	C57—N5	1.4692 (15)
C23—H23	0.9500	C57—H571	0.9900
C24—C25	1.3835 (16)	C57—H572	0.9900
C24—H24	0.9500	N5—C51	1.4199 (14)
C25—C26	1.3908 (15)	N5—H5	0.922 (11)
C25—H25	0.9500	C51—C56	1.3884 (16)
C26—H26	0.9500	C51—C52	1.3940 (16)
N3—C4	1.3389 (13)	C52—C53	1.3879 (15)
C4—N4	1.3705 (14)	C52—H52	0.9500
C4—C5	1.4173 (15)	C53—C54	1.3726 (17)
N4—C41	1.4091 (15)	C53—H53	0.9500
N4—H4	0.869 (11)	F5—C54	1.3694 (13)
C41—C42	1.3932 (15)	C54—C55	1.3688 (16)
C41—C46	1.3980 (15)	C55—C56	1.3876 (16)
C42—C43	1.3820 (16)	C55—H55	0.9500
C42—H42	0.9500	C56—H56	0.9500
C43—C44	1.3905 (16)	C6—C61	1.5043 (16)
C43—H43	0.9500	C61—H611	0.9800
C44—C45	1.3935 (16)	C61—H612	0.9800
C44—C47	1.5081 (17)	C61—H613	0.9800
			
C2—N1—C6	116.60 (9)	H472—C47—H471	109.5
N1—C2—N3	126.73 (9)	C46—C45—C44	122.35 (10)
N1—C2—C21	116.67 (9)	C46—C45—H45	118.8
N3—C2—C21	116.55 (10)	C44—C45—H45	118.8
C22—C21—C26	118.70 (10)	C45—C46—C41	119.72 (10)
C22—C21—C2	120.54 (10)	C45—C46—H46	120.1
C26—C21—C2	120.73 (10)	C41—C46—H46	120.1
C23—C22—C21	120.65 (11)	C6—C5—C4	116.38 (9)
C23—C22—H22	119.7	C6—C5—C57	123.37 (10)
C21—C22—H22	119.7	C4—C5—C57	120.15 (9)
C24—C23—C22	120.24 (11)	N5—C57—C5	111.50 (9)
C24—C23—H23	119.9	N5—C57—H571	109.3
C22—C23—H23	119.9	C5—C57—H571	109.3
C25—C24—C23	119.80 (11)	N5—C57—H572	109.3
C25—C24—H24	120.1	C5—C57—H572	109.3
C23—C24—H24	120.1	H571—C57—H572	108.0
C24—C25—C26	120.13 (11)	C51—N5—C57	116.18 (9)
C24—C25—H25	119.9	C51—N5—H5	110.7 (7)
C26—C25—H25	119.9	C57—N5—H5	109.2 (7)
C25—C26—C21	120.47 (10)	C56—C51—C52	118.76 (10)
C25—C26—H26	119.8	C56—C51—N5	120.78 (10)
C21—C26—H26	119.8	C52—C51—N5	120.45 (10)
C4—N3—C2	116.05 (9)	C53—C52—C51	120.76 (11)
N3—C4—N4	119.36 (10)	C53—C52—H52	119.6
N3—C4—C5	122.42 (9)	C51—C52—H52	119.6
N4—C4—C5	118.22 (9)	C54—C53—C52	118.55 (11)
C4—N4—C41	130.21 (9)	C54—C53—H53	120.7
C4—N4—H4	114.2 (7)	C52—C53—H53	120.7
C41—N4—H4	114.6 (7)	C55—C54—F5	118.87 (10)
C42—C41—C46	118.41 (10)	C55—C54—C53	122.38 (10)
C42—C41—N4	116.76 (10)	F5—C54—C53	118.75 (10)
C46—C41—N4	124.71 (10)	C54—C55—C56	118.76 (11)
C43—C42—C41	120.88 (10)	C54—C55—H55	120.6
C43—C42—H42	119.6	C56—C55—H55	120.6
C41—C42—H42	119.6	C55—C56—C51	120.78 (11)
C42—C43—C44	121.60 (10)	C55—C56—H56	119.6
C42—C43—H43	119.2	C51—C56—H56	119.6
C44—C43—H43	119.2	N1—C6—C5	121.77 (10)
C43—C44—C45	117.02 (10)	N1—C6—C61	114.84 (9)
C43—C44—C47	121.06 (10)	C5—C6—C61	123.38 (9)
C45—C44—C47	121.89 (10)	C6—C61—H611	109.5
C44—C47—H473	109.5	C6—C61—H612	109.5
C44—C47—H472	109.5	H611—C61—H612	109.5
H473—C47—H472	109.5	C6—C61—H613	109.5
C44—C47—H471	109.5	H611—C61—H613	109.5
H473—C47—H471	109.5	H612—C61—H613	109.5
			
C6—N1—C2—N3	1.40 (15)	C44—C45—C46—C41	−0.85 (16)
C6—N1—C2—C21	−175.79 (9)	C42—C41—C46—C45	−0.49 (14)
N1—C2—C21—C22	−10.66 (14)	N4—C41—C46—C45	−176.22 (9)
N3—C2—C21—C22	171.85 (9)	N3—C4—C5—C6	−1.67 (14)
N1—C2—C21—C26	167.46 (9)	N4—C4—C5—C6	177.97 (9)
N3—C2—C21—C26	−10.03 (14)	N3—C4—C5—C57	175.03 (9)
C26—C21—C22—C23	1.20 (16)	N4—C4—C5—C57	−5.32 (14)
C2—C21—C22—C23	179.36 (10)	C6—C5—C57—N5	−116.78 (12)
C21—C22—C23—C24	−0.62 (17)	C4—C5—C57—N5	66.75 (13)
C22—C23—C24—C25	−0.41 (17)	C5—C57—N5—C51	173.10 (9)
C23—C24—C25—C26	0.82 (17)	C57—N5—C51—C56	−132.72 (11)
C24—C25—C26—C21	−0.22 (16)	C57—N5—C51—C52	48.62 (14)
C22—C21—C26—C25	−0.78 (15)	C56—C51—C52—C53	1.29 (15)
C2—C21—C26—C25	−178.94 (10)	N5—C51—C52—C53	179.98 (10)
N1—C2—N3—C4	−0.86 (15)	C51—C52—C53—C54	−0.79 (16)
C21—C2—N3—C4	176.34 (9)	C52—C53—C54—C55	−0.40 (16)
C2—N3—C4—N4	−178.66 (9)	C52—C53—C54—F5	178.85 (9)
C2—N3—C4—C5	0.98 (14)	F5—C54—C55—C56	−178.21 (9)
N3—C4—N4—C41	−9.67 (16)	C53—C54—C55—C56	1.04 (16)
C5—C4—N4—C41	170.67 (10)	C54—C55—C56—C51	−0.50 (16)
C4—N4—C41—C42	166.24 (10)	C52—C51—C56—C55	−0.64 (15)
C4—N4—C41—C46	−17.97 (17)	N5—C51—C56—C55	−179.32 (10)
C46—C41—C42—C43	1.63 (14)	C2—N1—C6—C5	−2.09 (14)
N4—C41—C42—C43	177.70 (9)	C2—N1—C6—C61	177.06 (9)
C41—C42—C43—C44	−1.47 (15)	C4—C5—C6—N1	2.23 (14)
C42—C43—C44—C45	0.12 (15)	C57—C5—C6—N1	−174.35 (9)
C42—C43—C44—C47	−178.02 (10)	C4—C5—C6—C61	−176.85 (9)
C43—C44—C45—C46	1.03 (15)	C57—C5—C6—C61	6.56 (15)
C47—C44—C45—C46	179.16 (10)		

### Hydrogen-bond geometry (Å, º)

*Cg*1 is the centroid of the N1/C2/N3/C4–C6 ring.

**Table:**

*D*—H···*A*	*D*—H	H···*A*	*D*···*A*	*D*—H···*A*
N4—H4···N5	0.869 (11)	2.294 (11)	2.9820 (18)	136.2 (9)
C57—H571···F5^i^	0.99	2.54	3.440 (2)	151
C57—H572···*Cg*1^ii^	0.99	2.60	3.467 (7)	147

Symmetry codes: (i) −*x*, −*y*+1, −*z*+2; (ii) −*x*, −*y*+1, −*z*+1.
